# Comparison of Different Aliphatic Polyester-Based Microparticles as Protein Delivery Systems

**DOI:** 10.3390/polym17192676

**Published:** 2025-10-03

**Authors:** Viktor Korzhikov-Vlakh, Ekaterina Sinitsyna, Mariia Stepanova, Evgenia Korzhikova-Vlakh, Tatiana Tennikova

**Affiliations:** 1 Institute of Chemistry, Saint-Petersburg State University, 198504 St. Petersburg, Russia; kat_sinitsyna@mail.ru (E.S.); e.korzhikova-vlakh@spbu.ru (E.K.-V.); tennikova@mail.ru (T.T.); 2 Branch of Petersburg Nuclear Physics Institute Named by B.P. Konstantinov of National Research Center “Kurchatov Institute”—Institute of Macromolecular Compounds, 199004 St. Petersburg, Russia; maristepanova@gmail.com

**Keywords:** aliphatic polyesters, ring-opening polymerization, microparticles, protein encapsulation, drug release

## Abstract

The utilization of encapsulated biopharmaceuticals, including peptides and proteins, has grown substantially in recent years. In this study, the influence of aliphatic polyester physicochemical properties, specifically crystallinity and hydrophobicity, on the development of protein-loaded microparticles was investigated. A series of polyesters, namely amorphous PDLLA and semicrystalline PLLA, PCL, and PPDL, were synthesized via chemical and enzymatic ring-opening polymerization. Bovine serum albumin (BSA)-loaded microparticles were fabricated using a water-in-oil-in-water (*w*/*o*/*w*) double emulsion solvent evaporation method. The size of microparticles obtained was determined by scanning electron microscopy and dynamic light scattering methods. The enzymatic degradation of the polymer microparticles was assessed through incubation in a lipase-containing buffer solution. BSA and α-chymotrypsin (ACHT) were used as model proteins for the preparation of encapsulated polymer microspheres and comparison of their characteristics and properties. Protein encapsulation efficacy, release rate, and enzyme activity retained after encapsulation were evaluated and compared for selected aliphatic polyesters. The release profiles were processed with the use of various mathematical models to reveal the possible mechanism(s) of protein release.

## 1. Introduction

The treatment and prevention of various diseases, e.g., infectious diseases, cancer, immune diseases, etc., often require the long-term maintenance of therapeutic concentrations of the respective drug substances in the patient’s body [1]. This task can be successfully accomplished by utilizing various formulations that provide sustained release of drug substances. In this regard, the last decade has been marked by an increasing interest in the use of drug delivery systems [2,3,4]. Small compounds [5,6], oligomeric molecules such as peptides [7] and oligonucleotides [8], and biological macromolecules, such as proteins [9,10] and nucleic acids [11], can be used as drugs. In contrast to small molecules, peptides, and nucleic acids, proteins are more complicated compounds that have a certain spatial folding that affects the biological function of the biomacromolecule. Currently, the use of proteins in medicine for the treatment of various diseases (from viral infections to genetic diseases) is extremely promising and has already found application in clinical practice [12,13,14].

The use of polymeric particles in vivo imposes certain limitations on the choice of polymers. Obviously, they should be biocompatible and preferably biodegradable. The latter is not only a way to eliminate particles from the body, but also a way to control the release of biologically active molecules from polymer particles by regulating the composition and structure of the polymer. Among the various polymers used in drug delivery systems, aliphatic polyesters [15] such as poly(lactic acid) (PLA) [16] and poly(lactide-co-glycolide) (PLGA) [17] have attracted particular attention. These polymers are authorized by American and European regulatory agencies for in vivo use.

Currently, numerous studies are devoted to the micro- and nanoencapsulation of small-molecule drugs within aliphatic polyesters [18,19,20,21,22]. Techniques such as single emulsion [23], double emulsion [24], and nanoprecipitation [25] are widely used to produce drug-encapsulated micro- and nanoparticles, depending on the drug’s physicochemical properties. While highly hydrophobic polyesters such as PCL achieve high encapsulation efficiency for hydrophobic drugs, hydrophilic drugs pose a challenge, often resulting in low encapsulation efficiency and a significant initial burst release due to their localization near the particle surface or within aqueous pores. However, in addition to hydrophobicity, the properties of drug delivery systems also depend on polymer crystallinity. This was demonstrated by Korzhikov et al., who developed risperidone-loaded microparticles from various polyesters, including poly(L-lactic acid) (PLLA), poly(D,L-lactic acid) (PDLLA), PLGA, poly(ε-caprolactone) (PCL), and poly(ω-pentadecalactone) (PPDL) [15]. Contrary to expectations, the drug release rate was primarily governed by molecular diffusion through porous structures in microparticles formed from semicrystalline polymers, rather than by overall polymer hydrophobicity.

While the encapsulation of low-molecular-weight drugs is well-studied, the encapsulation of proteins in aliphatic polyester-based systems remains less explored, with only a few known examples [26,27,28,29,30,31,32]. Unlike small molecules that are loaded by the single emulsion (water/oil, or *w*/*o*) method, which involves dissolving the drug into an organic phase not suitable for proteins, the water-in-oil-in-water (*w*/*o*/*w*) double emulsion method is commonly used for loading proteins. For instance, the *w*/*o*/*w* method was used by Tobio et al. to prepare PLA and PEG-*b*-PLA particles loaded with tetanus toxoid (a potent exotoxin produced by *Clostridium tetani*) [26]. Using the same technique, Huang et al. developed a method for the preparation of PLGA nanoparticles loaded with bovine serum albumin (BSA) [28]. Recently, Gaur et al. reported the preparation of the same formulation, namely BSA-loaded PLGA nanoparticles, but additionally covalently modified with chitosan [30]. In addition to the *w*/*o*/*w* technique, the emulsion/spray-drying technique can be successfully applied to prepare PLGA-based microparticles loaded with recombinant tuberculosis antigen (TB10.4-Ag85B) for pulmonary delivery as a vaccine to prevent tuberculosis [32]. The differences in the immunization behavior of PLA-based micro- and nanoparticles bearing the same protein were recently investigated by Sakhabeev et al. [33,34]. In turn, Bilati et al. reported the encapsulation of three model proteins (tetanus toxoid, lysozyme, and insulin) into PLGA and PDLLA particles using modified double emulsion and nanoprecipitation techniques [31]. While optimization of the solvent system in the double emulsion technique yielded high encapsulation efficiency for all proteins, the nanoprecipitation method was particularly effective, generating smaller, more homogeneous particles and achieving the highest efficacy for lysozyme.

Despite existing studies on the encapsulation of proteins into PLA and PLGA micro-and nanoparticles, there is currently no comparative study examining the effect of the hydrophobicity and crystallinity of the aliphatic polyester on protein encapsulation efficacy and release rate. Considering the current state of the art, the goal of the present study was to prepare the protein-loaded microparticles based on aliphatic polyesters with different hydrophobicity and crystallinity. In this regard, four aliphatic polyesters were used in the study, namely, PDLLA, PLLA, PCL, and PPDL. BSA and α-chymotrypsin (ACHT) were used as model proteins for the preparation of encapsulated polymer microspheres and comparison of their characteristics and properties.

## 2. Materials and Methods

### 2.1. Materials

The monomers, namely ω-pentadecalactone (PDL, ≥98%), ε-caprolactone (CL, 97%), and *L*-lactide (*L*-LA, 98%) and *rac*-lactide (rLA, 99%), were products of Merck (Darmstadt, Germany). All surfactants used as stabilizers for microparticles—sodium dodecyl sulfate (SDS), soybean lecithin, didodecyldimethylammonium bromide (DMAB), Lutrol F-68, and poly-*N*-vinylpyrrolidone (PVP, 40,000)—were purchased from Sigma-Aldrich (Schnelldorf, Germany). Catalysts for ring-opening polymerization, namely, stannous(II) 2-ethylhexanoate (Sn(Oct)_2_, 92.5–100%), and acrylic resin immobilized *Candida antarctica* lipase *B* (CALB, Novozyme-435), possessing specific activity ≥10,000 U/g, were the product of Merck (Darmstadt, Germany). Amano Lipase from *Pseudomonas fluorescens* (≥20,000 U/g, Sigma-Aldrich, Schnelldorf, Germany) was used for the polymer degradation study.

The organic solvents (toluene, methanol, ethanol, acetone, ethyl acetate, dichloromethane, etc.) used for the polymer synthesis and purification, as well as for microparticles production, were purchased from Vekton (St. Petersburg, Russia). Prior to use, all organic solvents were purified by distillation using standard methods. Toluene was additionally dried over calcium hydride before distillation.

Salts for buffer preparation were of analytical grade and were also acquired from Vekton (St. Petersburg, Russia). The proteins—bovine serum albumin (BSA, ≥98%) and chymotrypsin from bovine pancreas (ACHT), as well as substrate *N*-benzoyl-*L*-tyrosine *p*-nitroanilide (BTpNA, ≥98%)—were products of Sigma-Aldrich (Schnelldorf, Germany).

### 2.2. Methods

#### 2.2.1. Synthesis and Characterization of Polymers

PLLA and PDLLA were synthesized using ring-opening polymerization *in bulk* using St(Oct)_2_ as a catalyst. The monomer to catalyst ratio ([M]/[SnOct_2_]) was 1000. The reaction was conducted in vacuum-processed Schlenk tubes at 120 °C for 6 h. The polymer was dissolved in a small amount of chloroform and precipitated into cold methanol. Purification was achieved by two additional dissolution/precipitation cycles in chloroform/cold methanol. Finally, the polymers were dried under vacuum at room temperature.

The synthesis of PCL and PDDL by enzyme-mediated ring-opening polymerization was similar to the previously published procedure [35]. The reaction was performed in dry toluene. A typical procedure for enzyme-mediated ROP is briefly described below. To a mixture of 1.5 g of PDL and 150 mg of CALB enzyme (monomer/enzyme = 10, wt/wt), 2 mL of toluene was added dropwise under vigorous stirring and argon purging. The synthesis was carried out at 100 °C for 2 h for PDL and 5 h for CL polymerization. After completion of the reaction, the mixture was cooled, and the resulting sample was dissolved in chloroform. The enzyme immobilized on the acrylic resin was removed from the polymer solution by filtration using a Schott filter. The polymer solution was then concentrated via rotary evaporation and precipitated into pre-cooled methanol. The polymers were purified and dried using the same procedure as for PLLA/PDLLA.

The polymers obtained were characterized by size-exclusion chromatography (SEC) using a Shimadzu chromatographic system (Kyoto, Japan), consisting of an LC-10AD pump and a RID-10A refractometric detector, supplied with a Waters Styragel HMW6E analytical column. Shimadzu LC Solution 1.24SP1 GPC software (Kyoto, Japan) was used for data processing. The analysis was performed in chloroform as the mobile phase using sample solutions with concentrations of 0.15–0.25 wt% depending on the expected molecular weight. Sample volume was 50 µL valve loop, and elution rate was 0.3 mL/min. The calculation of molecular weight and dispersity was performed regarding a preliminary plotted calibration curve (polystyrene standards). In addition, the intrinsic viscosity of polymer solutions in chloroform was determined using an Ostwald-type viscometer (capillary diameter: 0.37 mm; Neva-Reactiv, St. Petersburg, Russia), which was thermostatically controlled by a LOIP LT-912 cryothermostat (LOIP, St. Petersburg, Russia).

#### 2.2.2. Preparation of Protein-Encapsulated Microparticles

Microparticles based on synthesized aliphatic polyesters of various hydrophobicity were prepared using the double emulsion method. For the preparation of water phase 1, BSA was dissolved in water to a concentration of 10 mg/mL. To obtain the organic (oil) phase, the polymer was thoroughly dissolved in an appropriate organic solvent (25 mg/mL) containing a surfactant system. Dichloromethane was used as the solvent for all polymers. In addition, a mixture of dichloromethane/acetone was also examined for PLLA. The organic phase also contained a surfactant system, which typically included 1% Lutrol F68 and 0. 5% DMAB. When dichloromethane/acetone was used as an organic phase for PLLA, DMAB was replaced with SDS. For some systems, lecithin was used together with the Lutrol F68 + DMAB system.

The prepared, cooled water phase 1 was then added dropwise over 2 min into the organic phase, while simultaneously treating it with an ultrasonic homogenizer at 10–40% power. The resulting emulsion was immediately added to water phase 2 (a 5% PVP solution in water) under dispersion with simultaneous stirring using a magnetic stirrer (700–1000 rpm) for 3 min. To reduce particle size, the resulting double emulsion was transferred into water phase 3 (a 0.5% PVP solution with 5% NaCl in water). The solvent was then immediately removed by rotary evaporation under mild conditions (water bath temperature of 8–10 °C, 1.5–3.0 h) to ensure preservation of the protein’s spatial structure.

After organic solvent removal and microparticle suspension formation, the particles were isolated by centrifugation for 10 min at 10,000 rpm on the same day. The resulting microparticle sediment was washed with water and centrifuged at least three times under the same speed and duration conditions. The supernatant and wash liquid were analyzed using the Lowry–Folin method to determine protein concentration. To obtain dry microparticles, the microparticle dispersion was frozen and lyophilized.

#### 2.2.3. Determination of Protein Encapsulation Efficiency

The amount of encapsulated protein was determined by calculating the difference between its initial concentration and the concentration measured in the supernatant and wash solutions (indirect method). In addition, determination of protein content in microparticles was performed by the particle dissolution method (direct method). In this case, 0.5 mL of 1 M NaOH solution was added to a microtube containing dried protein-loaded microparticles. The mixture was stirred under heating at 60 °C until complete dissolution. The resulting solution was transferred to an ultrafiltration cell (10 kDa membrane). The protein-containing solution, purified from hydrolyzed polymer fragments after repeated ultrafiltration with water, was diluted with water to a final volume of 6 mL and adjusted to neutral pH with 0.1 M HCl solution.

In all cases, protein concentration was quantified using the Lowry–Folin method with spectrophotometric analysis performed at 750 nm. The optical densities of the solutions were converted to concentrations using a previously constructed calibration curve for the same protein (BSA or ACHT). The total protein content in the analyzed system was then calculated by accounting for the solution volumes. The encapsulation efficiency (*EE*, %) was calculated using Equation (1):

(1) $$EE\;=\;\frac{Q_{en}}{Q_{o}}\cdot 100\%$$

where *Q*_0_ is an initial amount of protein used for encapsulation (mg); *Q_en_* is an amount of encapsulated protein (mg).

#### 2.2.4. Characterization of Microparticles

The morphology of the obtained samples was imaged using a Zeiss Supra 40VP (Oberkochen, Cermany) scanning electron microscope (SEM). The mean size of dry particles was estimated from SEM images using free ImageJ software (v.1.53n, Open access), developed by the National Institute of Health (Washington, DC, USA). For measurement of particle size distribution, a dynamic light-scattering (DLS, Zetasizer Nano ZS, Malvern, UK) instrument was used.

#### 2.2.5. Polymer Degradation Study

A sample (50 mg) of dry particles was placed into a plastic microtube and mixed with 1.0 mL of a degradation medium consisting of 0.01 M PBS (pH 7.4), 0.1% SDS, and 3.0 mg of Amano Lipase. At predetermined time intervals, the particles were centrifuged, washed with distilled water, freeze-dried, and weighed to determine the weight loss. The weight loss was calculated as follows (2):

(2) $$\mathrm{Weight}\mathrm{loss}\;=\;\frac{m_{t}}{m_{o}}\cdot 100\%$$

where *m_t_* is a mass of polymer microparticles at time point *t* (mg); *m_o_* is an initial mass of polymer microparticles (mg).

#### 2.2.6. Protein Release Study

The release study was performed using 5 mg of microparticles in 1.7 mL of 0.01 M phosphate-buffered saline (PBS), pH 7.4, at 37 °C. The percentage of drug release was calculated as the ratio of protein detected in the supernatant at each time point to the total loaded protein amount. At predetermined intervals, the particles were centrifuged (10,000 rpm, 10 min) and the supernatant was collected for protein analysis using the Lowry–Folin method. The released protein concentration was determined using a pre-established calibration curve. After each sampling, an equal volume of fresh buffer was added to maintain constant volume conditions, and the particles were returned to incubation at 37 °C until the next time point. The data were processed to generate a cumulative release profile, expressed as a percentage of release versus time.

#### 2.2.7. Enzyme Activity Assay

The enzymatic activity was studied using BTpNA as a substrate. Samples of native enzyme or enzyme released from microparticles and lyophilized were dissolved in Tris-HCl buffer (pH 7.8) at a concentration of 1 mg/mL. The substrate was initially dissolved in DMSO (10 mg/mL), followed by preparation of a series of substrate solutions in DMSO/buffer (50/50, *v*/*v*) with concentrations ranging from 0.05 to 2.4 mg/mL (final volume 1.5 mL). For the assay, the substrate solution was placed in a spectrophotometric cuvette, and the initial optical density (measured at time zero) was recorded. Then, 25 μL of enzyme solution was added to the substrate solution, and the optical density was measured after 10 min at 420 nm. The reaction velocity at substrate concentration *C* (*V_c_*, μmol·L^−1^·min^−1^) was calculated using Equation (3):

(3) $$V_{c}=\;\frac{A}{\varepsilon \cdot l\cdot t}$$

where *A* is the optical density of the solution after reaction, *l* is the cuvette thickness, *ε* is the extension of pNP, and *t* is the time of reaction.

The apparent values of Michaelis constant (*Kₘ*) and maximum reaction velocity (*Vₘₐₓ*) were determined using the Michaelis–Menten plot (*V_c_* vs. *C*) and its linearized form in double reciprocal coordinates (Lineweaver–Burk plot). Specific activity (*A_sp_*, µmol·min^−1^·mg^−1^) was calculated as follows (4):

(4) $$A_{sp}=\;\frac{V_{max}}{\nu \;\cdot m}$$

where *ν* is a reaction volume (L) and *m* is the mass of enzyme in the system (mg).

#### 2.2.8. Data Processing and Statistics

All experiments on the preparation of microparticles, their encapsulated forms, and their characterization were performed in triplicate. The data were processed using Excel software (MS Office 2021, Redmond, WA, USA) to calculate mean and standard deviation values. Statistical significance was analyzed using a *t*-test (GraphPad software, version Prism 10.1.2, La Jolla, CA, USA). *p* < 0.05 was considered a statistically significant difference.

## 3. Results and Discussion

### 3.1. Polymers and Their Characteristics

The aliphatic polyesters selected for this study demonstrate substantial variations in their physicochemical properties, particularly in terms of crystallinity and hydrophobicity. These differences directly affect the structural characteristics of the resulting microparticles, including their porosity and degradation behavior, thereby governing the drug release profile from the delivery system. The key differences between the selected aliphatic polyesters are summarized in Table 1. Among the aliphatic polyesters listed in Table 1, PDLLA is the only amorphous polymer, whereas PLLA, PCL, and PPDL are semicrystalline. The amorphous nature of PDLLA contributes to its lower hydrophobicity and faster degradation rate compared to semicrystalline polymers. For the three semicrystalline polyesters, crystallinity and hydrophobicity increase as follows: PLLA < PCL < PPDL, resulting in corresponding increases in degradation resistance.

In this study, PLLA and PDLLA were synthesized by ring-opening polymerization (ROP) of *L-* or *rac*-lactide, respectively, *in bulk* in the presence of stannous octoate(II) as a catalyst [36]. In contrast to small cyclic monomers like lactide, glycolide, and *β*-butyrolactone, larger lactones such as ε-caprolactone (CL) and pentadecalactone (PDL) can be efficiently polymerized via enzymatic catalysis [37]. Moreover, unlike PCL, which can be successfully synthesized using either chemical or biocatalytic methods, PPDL can only be efficiently obtained via enzyme-catalyzed ROP. Notably, immobilized *Candida antarctica* lipase B (CALB) offers a robust and environmentally friendly catalytic approach. The CALB immobilized on acrylic resin offers both catalytic efficiency and practical advantages, including easy filtration recovery and reusability. In this study, the commercial biocatalyst Novozyme-435 (immobilized CALB) was utilized for the enzymatic ROP of CL and PDL in toluene to synthesize PCL and PPDL [15].

**Table 1:** Chemical structures and physicochemical properties of the selected aliphatic polyesters.

Polymer Structure		Physicochemical Properties		
		Hydrophobicity	Crystallinity	Degradation
	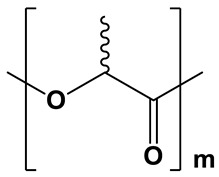	Moderately hydrophobic; water contact angle =68–76° [38,39,40]	Amorphous; XRD: the region 2θ = 15.0–30.0° contains only a pronounced “amorphous halo” [15]	Relatively fast degradation; 3–6 months [41]
PDLLA				
PLLA	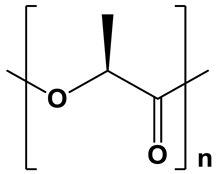	Hydrophobic; water contact angle =74–85° [42,43,44]	Semicrystalline; degree of crystallinity up to 40% (50%, thermal treatment) [45,46,47,48]; XRD: two sharp signals at 2θ = 16.6° and 19.0° [15]	Moderate degradation; From 6 months to 2 years, depending on the crystallinity degree and molecular weight [41,49]
	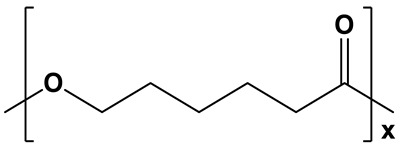	Highly hydrophobic; water contact angle =78–95° [50,51,52]	Semicrystalline;degree of crystallinity from 39 to 69% [53,54,55]; XRD: two sharp signals at 2θ = 21.3° and 23.6° [15,55]	Long degradation; from 1 to 3 years [53,56]
PCL				


PPDL	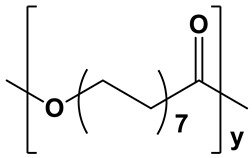	Extremely hydrophobic; water contact angle ≥93° [57]	Semicrystalline; degree of crystallinity from 54 to 74% [58,59,60,61]; XRD: two sharp signals at 2θ = 21.4° and 23.9° [15]	Long degradation;up to several years [58,61,62]

The molecular weight characteristics of the synthesized polymers are summarized in Table 2. The polymerization conditions were selected to achieve a degree of polymerization (*DP*) of approximately 100. Due to variations in the molecular weights of the monomer units comprising the polyesters, the molecular weights of the resulting polymers differed accordingly, as expected.

### 3.2. Preparation and Characterization of Protein-Loaded Microparticles

**Figure 1:**
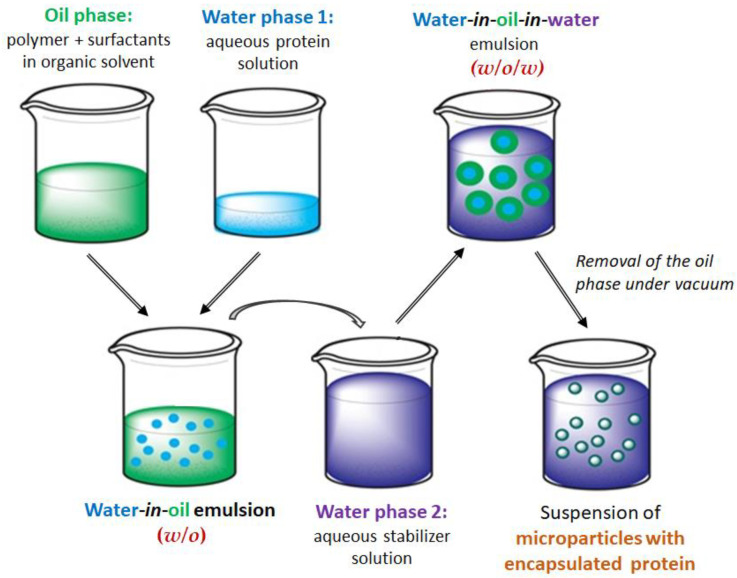
Scheme for the preparation of protein-loaded microparticles by the double emulsion (*w*/*o*/*w*) method followed by organic solvent evaporation.

The BSA-loaded aliphatic polyester microparticles were prepared using a water-in-oil-in-water (*w*/*o*/*w*) double emulsion method (Figure 1). In this process, the primary emulsion was formed by dispersing an aqueous protein solution into an organic phase consisting of an aliphatic polyester and surfactants dissolved in a water-immiscible solvent, yielding a water-in-oil (*w*/*o*) emulsion. This emulsion was then inverted by adding a large excess of an aqueous stabilizer solution, creating a *w*/*o*/*w* double emulsion. The subsequent gradual removal of the organic solvent via evaporation led to the formation of a suspension of polymer microparticles encapsulating the protein macromolecules.

Surfactants are required to prevent the polymer from precipitating during emulsification. Their role is to lower the surface tension at the water-oil interface, which prevents the emulsion from breaking down and stops the polymer from forming a solid phase. The selection of surfactants was based on their hydrophilic–lipophilic balance (HLB) values. High-HLB surfactants (>20), such as Lutrol-F68 and DMAB, were used to stabilize the oil-in-water (*o*/*w*) interface, as they are known to effectively stabilize direct *o/w* emulsions. Additionally, the role of lecithin, a low-HLB surfactant (HLB 4–7), was investigated due to its potential to stabilize the primary *w*/*o* emulsion and maintain the biphasic droplet structure [63]. Due to its low HLB value, lecithin is a highly suitable biological emulsifier for forming *w*/*o* emulsions [64]. PVP was employed as a stabilizer in the *w*/*o*/*w* double emulsion to stabilize the outer aqueous phase and protect the colloidal system.

**Figure 2:**
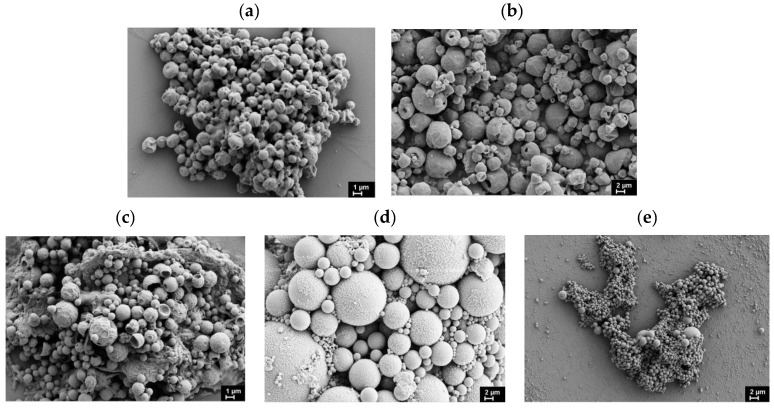
SEM images of microparticles loaded with BSA using the double emulsion method: (**a**) PLLA (dichloromethane/acetone + Lutrol F68 + SDS), (**b**) PDLLA (dichloromethane + Lutrol F68 + DMAB + lecithin), (**c**) PCL (dichloromethane + Lutrol F68 + DMAB + lecithin), (**d**) PPDL (dichloromethane + Lutrol F68 + DMAB), and (**e**) PPDL (dichloromethane + Lutrol F68 + DMAB + lecithin).

The characteristics of the resulting microparticles under different preparation conditions are summarized in Table 3. Particle size and morphology were analyzed using both SEM and DLS, revealing spherical microparticles with diameters in the micrometer range (Table 3, Figure 2). The average particle sizes obtained from SEM and DLS measurements showed good agreement. The data reveal that while PLLA, PDLLA, and PCL yield microparticles with comparable average sizes, a significant size increase is observed for PPDL-based microparticles. This size difference likely results from PPDL’s significantly higher hydrophobicity compared to the other polymers, promoting aggregation of initially formed particles into larger structures. Our findings suggest that polymer hydrophobicity only negligibly affects particle size within a moderate range of changes in hydrophobicity (PDLLA, PLLA, PCL). However, when hydrophobicity exceeds a critical threshold (as with PPDL), alternative stabilization strategies become necessary to prevent particle aggregation. Introduction of lecithin as a stabilizer in the organic phase significantly reduced the average particle diameter, confirming its efficacy in controlling particle size (Table 3). Notably, this size-reducing effect was most pronounced for the highly hydrophobic PPDL, suggesting that lecithin’s stabilizing mechanism is particularly effective for polymers with elevated hydrophobicity.

Previously published reports on amorphous PLGA describe protein-loaded microparticles with diameters of 0.5–1.5 µm [31], produced via a double emulsion method, and 2–4 µm, produced via an emulsion/spray-drying method [32]. These size ranges are quite close to our observations for amorphous PDLLA. In contrast, the encapsulation of BSA in PCL via double emulsion has been reported to yield larger particles (6–34 µm) [65], which significantly exceed the 2–3 µm microparticles achieved for PCL in our work.

**Figure 3:**
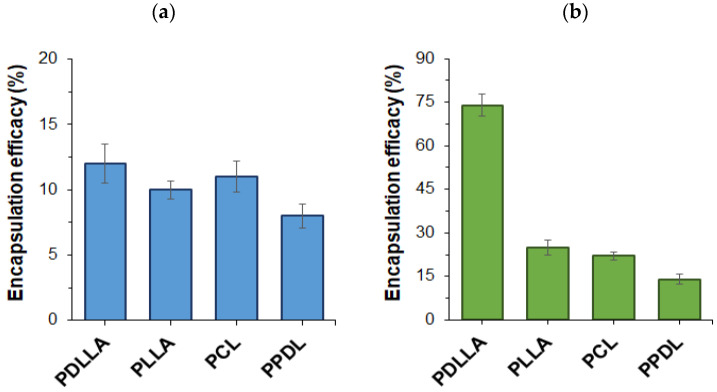
Effect of aliphatic polyester on BSA encapsulation efficacy in the presence of different surfactant systems: (**a**) Lutrol F68 + DMAB in dichloromethane as organic phase for PDLLA, PCL, and PPDL, and Lutrol F68 + SDS in dichloromethane/acetone for PLLA; (**b**) Lutrol F68 + DMAB + lecithin in dichloromethane for all polymers.

Given that the polymers employed for BSA encapsulation varied in both hydrophobicity and crystallinity, their impact on protein encapsulation efficiency (*EE*) was evaluated. As shown in Figure 3a, the choice of polyester had only a slight influence on BSA encapsulation efficacy when Lutrol F68 and DMAB were used as the stabilizers for the microparticles. In this case, encapsulation efficacy remained relatively consistent across all polymers and ranged from 8 to 12%. For PLLA, the encapsulation efficiency was quite low (4–6%) when using dichloromethane as the organic phase, but increased significantly with the application of a dichloromethane/acetone mixture (10%) as the organic phase (Figure 3a).

The effect of adding lecithin to the organic phase is illustrated in Figure 3b. The results demonstrate a consistent increase in protein encapsulation efficacy when lecithin was employed as a co-surfactant, revealing clear structure–property relationships. This supports the validity of the stabilizer selection strategy for preserving the biphasic droplet structure. The most significant improvement in encapsulation efficacy was observed when amorphous PDLLA was used as the polymer to prepare microparticles. This may be attributed to the less ordered arrangement of PDLLA macromolecules, providing more space for protein encapsulation. In contrast, the lower encapsulation efficacy observed with PLLA, PCL, and PPDL likely stems from their semicrystalline nature. Notably, when the most hydrophobic polymer, PPDL, was used, encapsulation efficacy decreased further. This can be explained by PPDL’s limited solubility in the organic phase, which increases the likelihood of polymer precipitation during suspension formation and solvent evaporation, ultimately reducing protein encapsulation efficiency.

The high protein encapsulation efficacy observed for amorphous PDLLA is consistent with data reported for amorphous PLGA, which ranges from 25% to 96% depending on the organic phase and protein used [31]. Conversely, the literature data on the encapsulation of proteins in semicrystalline PLLA confirm significantly lower encapsulation parameters, which are also consistent with our data. In particular, Conway et al. reported encapsulation efficiencies of 3–7% for lactoglobulins and about 8% for BSA in PLLA-based microparticles [66]. The previously reported encapsulation efficiency for BSA in PCL-based microparticles was 17–25%, which is very close to our optimized value of 22%.However, the PCL microparticles developed in this study are several times smaller than those previously reported [65].

### 3.3. Particle Biodegradation and Protein Release

**Figure 4:**
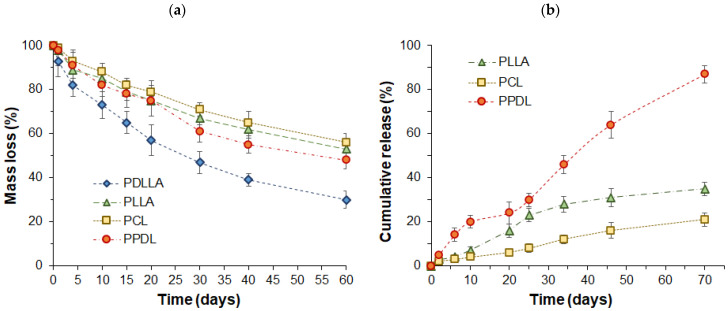
Enzymatically catalyzed degradation of various polyester microparticles (**a**) and plots of BSA release from semicrystalline polyesters (**b**). The study involved PLLA, PDLLA, and PCL microparticles with a size of ~2–3 µm, and PPDL microparticles with a size of 1 µm.

Biodegradability is one of the key characteristics affecting the rate of drug release and is strongly dependent on the structure and physicochemical properties of the polymer. The dependence of particle mass loss on time during enzyme-catalyzed degradation is presented in Figure 4a. As expected, microparticles composed of amorphous PDLLA exhibited the fastest degradation rate. This can be attributed to the polymer’s high permeability to water molecules, facilitating rapid hydrolytic degradation. In contrast, semicrystalline polymers degraded at comparable rates, although their behavior was governed by two competing factors, namely, water sorption and microparticles’ surface area. Specifically, the slightly faster degradation of hydrophobic PPDL, despite its low affinity for the aqueous phase, may be attributed to its smaller particle size, which results in a higher surface area for contact with water and enzyme molecules.

According to the literature, drug release from semicrystalline polyester particles proceeds faster than from particles composed of amorphous polymers [35]. Based on this, protein-loaded microparticles made from three semicrystalline polymers were selected for the release study. The study of BSA release from microparticles revealed that the release rate depended on the type of aliphatic polyester used (Figure 4b). The most rapid release occurred with PPDL-based microparticles, which have the highest degree of crystallinity. In this case, 87% of the protein was released within two months. The release profile exhibited two distinct phases: an initial burst release, followed by a gradual sustained release. The burst release is attributed to protein desorption from the particle surface and diffusion from pores. The subsequent gradual release may result from protein diffusion through surface pores, which are more prevalent in highly crystalline polymers, as well as polymer degradation, which enlarges pores and facilitates protein diffusion from the particle core. Additionally, as previously noted, PPDL also exhibited the lowest encapsulation efficiency. In contrast, PLLA and PCL, which had similar encapsulation efficiencies, showed slower BSA release. Between these two polymers, PLLA microparticles demonstrated a faster release rate, whereas the more hydrophobic PCL exhibited a more gradual release profile.

The faster protein release from PPDL microparticles compared to those of other semicrystalline polymers is not a consequence of their 2-3 times smaller particle size. This is supported by a reported study on the small drug risperidone, which also showed the fastest release from PPDL among various aliphatic polyester microparticles, even when the PPDL particles were larger (2 µm) than others in the 0.5–1 µm range [15]. This fact indicates that the primary factor influencing the release rate is not the size of the PPDL microparticles, but rather their very high crystallinity, which affects the formation of a highly porous structure.

Regarding the release of proteins, literature data is available on the release of BSA from PLLA-based microparticles. According to published data, the release reaches 80% after two weeks, whereas in our study, it was only about 15% over the same period. The faster release reported in the literature can be attributed to the smaller microparticle size (~1 µm) and the considerably lower molecular weight PLLA (2000 Da) used for their fabrication [66], in contrast to the materials utilized in this study. According to the literature data on protein release from PCL-based microparticles with a diameter of 24 µm, the cumulative release reached approximately 28% after one month [65]. In our case, this value was lower for the same period, amounting to about 12%. However, as with the PLLA-based microparticles, the characteristics of the microparticles and PCL used in our study differed significantly from those reported by other authors.

The data in Table 4 present the goodness-of-fit (R^2^ values) for various mathematical models applied to protein release profiles from three different polyester microparticles, PLLA, PCL, and PPDL. The analysis was performed on both the entire release curve (70 days) and on the first 20 days of release.

**Figure 5:**
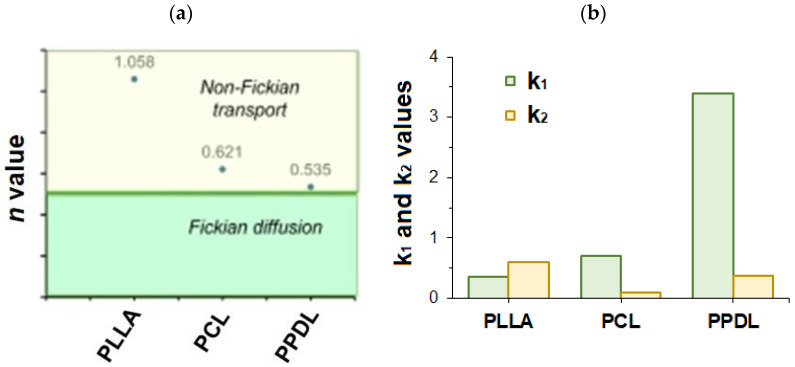
Results of approximation of the protein release profiles with application of various mathematical models: (**a**) comparison of *n* parameter obtained from the Korsemeyer–Peppas model for different systems under investigation; (**b**) comparison of *k*_1_ and *k*_2_ parameters, obtained from the Peppas–Sahlin model and showing the impact of diffusion and polymer relaxation, respectively.

In the case of protein release from PLLA microparticles, the best-fitting release models were found to be the Korsmeyer–Peppas (R^2^ = 0.9940), Peppas–Sahlin (R^2^ = 0.9935), and Hixson–Crowell (R^2^ = 0.9921) models. The high value of the release exponent *n* in the Korsmeyer–Peppas model (Figure 5a, *n* = 1.219 > 0.89) suggests a Super Case-II transport mechanism, which is dominated by polymer relaxation and erosion rather than simple diffusion. This is supported by the good fit of the Hixson–Crowell model, which describes erosion-controlled release. For PCL-based microparticles, the first-order (R^2^ = 0.9942), Hopfenberg (R^2^ = 0.9942), and Weibull (R^2^ = 0.9942) models fit exceptionally well. The Korsmeyer–Peppas model also fits well (R^2^ = 0.9905) with an exponent *n* = 0.630, which indicates anomalous (non-Fickian) transport—a combination of diffusion and erosion mechanisms. The excellent fit of the Hopfenberg model, which is explicitly designed for erosion-controlled release, suggests that surface erosion plays a significant role in this process. In the case of release from PPDL microparticles, the zero-order model provides the best fit for the full curve (R^2^ = 0.9926), indicating a constant release rate over time. This is a desirable release profile for many drug delivery applications. The Korsmeyer–Peppas model also fits well (R^2^ = 0.9885) with an exponent *n* = 0.557, which is close to 0.5 (Figure 5a), suggesting release is primarily governed by Fickian diffusion.

Based on the results of the mathematical processing of protein release profiles, one can conclude that the choice of polymer drastically alters the protein release mechanism. PLLA microparticles exhibit erosion-controlled release, PCL microparticles demonstrate a mixed diffusion-erosion mechanism, and PPDL microparticles provide a steady, diffusion-controlled release profile closest to the ideal zero-order kinetics. These conclusions are also confirmed by the ratio between *k*_1_ and *k*_2_ constants obtained from the Peppas–Sahlin model (Figure 5b). *k*_1_ is known as the diffusion constant, and *k*_2_ as the relaxation constant. When *k_1_* > *k_2_*, as in the case of PCL and PPDL, the release is governed by a diffusion mechanism. However, when the *k_1_*/*k_2_* ratio is close to 1 (the case of PLLA), it is evidence for anomalous transport in which both diffusion and relaxation are important.

Another important finding is that the best-fit model can change depending on the timeframe analyzed (e.g., full curve vs. first 20 days). This highlights that the release mechanism is not constant throughout the entire process. For example, an initial burst release (common in microparticles) can favor a first-order model in the short term, while a model like zero-order or Hopfenberg might better describe the longer-term, steady-state release phase. Overall, it could be concluded that the selection of a polymer for protein encapsulation predetermines the desired release profile for a specific therapeutic application.

### 3.4. Enzyme Encapsulation and Activity Study

To assess whether the released protein retained its biochemical activity, α-chymotrypsin (ACHT) was used as a model enzyme, given its well-characterized substrate cleavage reaction. ACHT was encapsulated into different polyesters (PDLLA, PLLA, PCL, and PPDL) via the double emulsion method. The encapsulation efficacy results revealed a trend: encapsulation efficacy decreased as polymer crystallinity increased (Table 5). Specifically, PPDL, the most crystalline polymer, showed the lowest encapsulation efficiency, while PLLA and PCL, which have lower crystallinity, demonstrated higher encapsulation values.

Compared to BSA, ACHT has a significantly lower molecular weight (the molecular weight of BSA is 66,000; the molecular weight of ACHT is 24,000). Despite the 2.7-fold difference in their molecular weights, BSA demonstrated higher encapsulation efficacy. This result can be explained by the difference in their hydrophobic amino acid content, which influences the presence of hydrophobic domains. Specifically, the hydrophobic amino acid content is approximately 50% for BSA versus 35% for ACHT. The difference in hydrophobicity generally explains the higher encapsulation efficacy observed for BSA compared to ACHT, despite its larger molecular weight and, consequently, protein globule volume.

**Figure 6:**
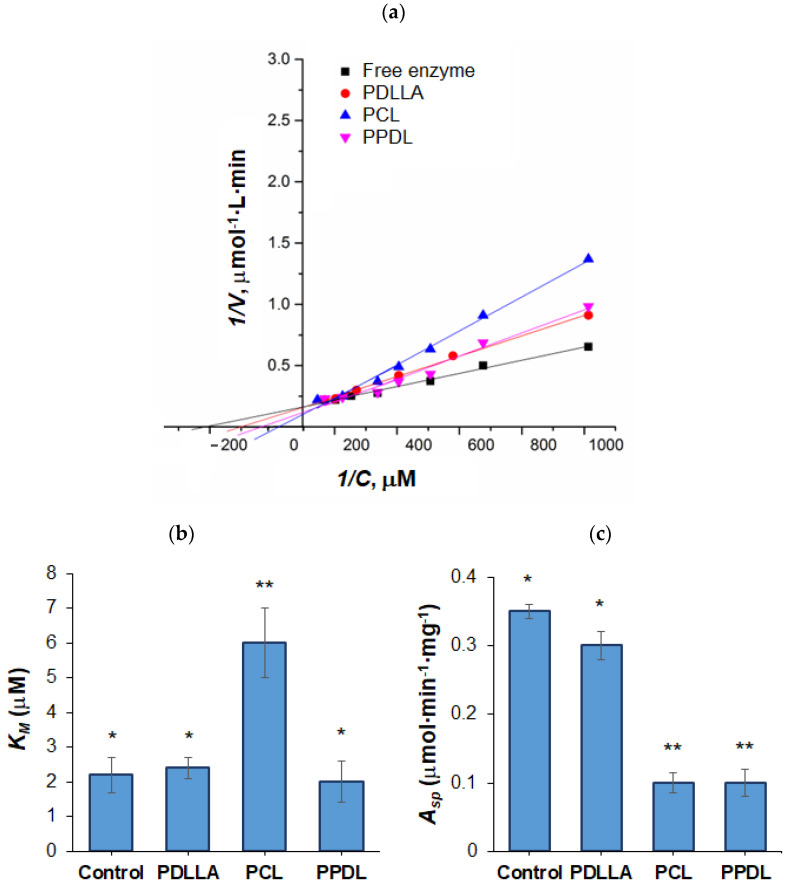
Lineweaver–Burk linearized Michaelis–Menten plots (**a**), Michaelis–Menten constants (**b**), and specific activity (**c**) for free ACHT and ACHT released from microparticles. Differences between values within the same group (* or **) are statistically non-significant (*p* > 0.05). In contrast, the difference between the * group and the ** group is statistically significant (*p* < 0.001).

In order to measure the activity of the encapsulated enzyme, enzyme release was conducted in phosphate-buffered saline solution (PBS, pH 7.4) over 14 days. The released enzyme was lyophilized, and its post-encapsulation activity was examined. ACHT activity was assessed via the hydrolysis of BTpNA into BT and pNA, where pNA is a chromogenic compound, which was quantified spectrophotometrically (Figure 6).

Reaction rates (*V*) were determined at varying substrate concentrations (*C*), and Michaelis–Menten kinetics plots (*V* vs. *C*) were built and linearized using double-reciprocal Lineweaver–Burk plots (Figure 6a) to derive Michaelis constants (*K_M_*) and specific activity (*A_sp_*) (Figure 6b,c). Notably, PLLA-encapsulated ACHT lost all activity, likely due to the dichloromethane/acetone solvent system (used to enhance encapsulation efficiency, as shown for BSA in Section 3.2), which may have disrupted the enzyme’s active site. In contrast, PDLLA-released ACHT retained near-native activity. PPDL encapsulation reduced activity but preserved *K_M_* value, while PCL performed the worst in maintaining catalytic properties. These results underscore the critical influence of polymer nature, solvent selection, and encapsulation conditions on preserving biological functionality.

## 4. Conclusions

Four aliphatic polyesters were synthesized by ring-opening polymerization, employing both chemical (for PDLLA and PLLA) and enzymatic (for PCL and PPDL) catalysis. The polymerization conditions were selected to achieve a comparable degree of polymerization across all polymers. This study demonstrated that high polymer hydrophobicity (PPDL) promoted the formation of larger microparticles; however, this effect was mitigated by the incorporation of the lecithin as a component of oil phase. While polymer type had a minor effect on encapsulation efficiency (*EE*) with standard surfactants, the addition of lecithin significantly improved encapsulation efficacy, with amorphous PDLLA achieving the highest values. Compared to amorphous PDLLA, the enzymatic degradation of semicrystalline polymers in the presence of lipase proceeded significantly more slowly. Based on the results of the mathematical processing of protein release profiles, one can conclude that the choice of polymer drastically alters the protein release mechanism. PLLA microparticles exhibit erosion-controlled release. In turn, PCL microparticles demonstrate a mixed diffusion-erosion mechanism, while PPDL microparticles provide a steady, diffusion-controlled release profile closest to the ideal zero-order kinetics. Furthermore, encapsulation of the model enzyme ACHT revealed that polymer choice and solvent system critically impacted the preservation of native enzymatic activity post-release. These findings highlight that tailoring polyester selection and formulation parameters is essential for optimizing protein encapsulation, release kinetics, and stability in biodegradable delivery systems.

## Figures and Tables

**Table 2:** Characteristics of aliphatic polyesters used in the study. Conditions: Synthesis of PLLA and PDLLA—ROP in bulk, [M]/[SnOct_2_] = 1000, temperature was 120 °C, reaction time was 6 h; synthesis of PCL and PPDL—ROP in toluene, monomer concentration was 86 wt%, [M]/[Lipase B] = 10, temperature was 100 °C, reaction time was 2 h for PDL and 5 h for CL polymerization.

Polymer	Yield, %	*M_n_* ^a^	*Ð* ^a^	*DP* ^b^	*η*, dL/g ^c^
PDLLA	68	8300	1.8	115	0.24
PLLA	66	8700	1.5	120	0.20
PCL	92	10,600	2.4	108	0.35
PPDL	88	19,000	1.9	85	0.90

**^a^** SEC in chloroform, calculated against polystyrene standards; **^b^** calculated by dividing the molecular weight of the polymer (*M_n_*) by the molecular weight of the monomer unit; **^c^** measured in CHCl_3_.

**Table 3:** Effect of oil phase composition on the size of BSA-loaded microparticles. Other conditions: water phase 1 – protein solution in water; water phase 2 – 5% PVP solution in water. Protein concentration was 10 mg/mL, polymer concentration in organic phase was 25 mg/mL. Concentration of surfactants in organic phase was constant: Lutrol F68 – 1%, DMAB/SDS – 0.5%, lecithin – 1%.

Organic (Oil) Phase			$\bar{D}$ (μm) ^a^	
Polymer	Solvent System	Surfactant System	SEM	DLS
PDLLA	Dichloromethane Dichloromethane	Lutrol F68 + DMAB Lutrol F68 + DMAB + lecithin	4.0 ± 1.9	2.8 ± 1.3
			3.1 ± 1.1	2.3 ± 0.8
PLLA	Dichloromethane Dichloromethane	Lutrol F68 + DMAB Lutrol F68 + DMAB + lecithin	3.0 ± 1.2	2.8 ± 0.9
			1.8 ± 0.5	1.0 ± 0.4
	Dichloromethane/acetone	Lutrol F68 + SDS	2.1 ± 0.6	1.8 ± 0.5
PCL	Dichloromethane Dichloromethane	Lutrol F68 + DMAB Lutrol F68 + DMAB + lecithin	3.0 ± 1.3	2.6 ± 0.8
			1.7 ± 0.6	2.0 ± 0.7
PPDL	Dichloromethane Dichloromethane	Lutrol F68 + DMAB Lutrol F68 + DMAB + lecithin	9.2 ± 3.9	10.1 ± 4.3
			0.9 ± 0.4	0.8 ± 0.3

**^a^** $\bar{D}$ ia the average particle diameter.

**Table 4:** Correlation coefficients and dissolution constants evaluated by fitting the protein release profiles from microparticles based on different polymers using standard mathematical models. The linearization curves are presented in Supplementary Materials (Figure S1).

Drug Release Model		Polymer Used for Microparticle Preparation		
		PLLA	PCL	PPDL
**Zero order** *F = k_0_* · *t*	*	0.9464 *k_0_ = 0.624*	**0.9931** *k_0_ = 0.321*	**0.9926** *k_0_ = 1.298*
	**	***0.9891*** *k_0_ = 0.778*	0.9594 *k_0_ = 0.337*	0.9276 *k_0_ = 1.433*
**First order** *F = 100* · *[1 − Exp(−k_1_* · *t)]*	*	0.9679 *k_1_ = 0.008*	**0.9942** *k_1_ = 0.004*	0.9756 *k_1_ = 0.020*
	**	***0.9866*** *k_1_ = 0.008*	0.9615 *k_1_ = 0.003*	0.9450 *k_1_ = 0.017*
**Higuchi** *F = k_H_* · *t^0.5^*	*	0.9713 *k_H_ = 4.174*	0.9616 *k_H_* = 2.084	0.9584 *k_H_* = 8.414
	**	0.9304 *k_H_* = 2.835	**0.9974** *k_H_* = 1.307	***0.9824*** *k_H_* = 5.577
**Korsmeyer-Peppas** *F = k_KP_* · *t^n^*	*	**0.9940** *k_KP_ = 0.440* *n = 1.219*	**0.9905** *k_KP_ = 0.989* *n = 0.630*	***0.9885*** *k_KP_ = 4.984* *n = 0.557*
	**	**0.9905** *k_KP_ = 0.662* *n = 1.058*	**0.9977** *k_KP_ = 1.239* *n = 0.521*	***0.9824*** *k_KP_ = 5.112* *n = 0.535*
**Hixson-Crowell** *F = 100* · *[1 − (1 − k_HC_* · *t)^3^]*	*	**0.9921** *k_HC_ = 0.003*	***0.9850*** *k_HC_ = 0.001*	0.9746 *k_HC_ = 0.005*
	**	***0.9875*** *k_HC_ = 0.003*	0.9608 *k_HC_ = 0.001*	0.9394 *k_HC_ = 0.005*
**Hopfenberg** *F = 100* · *[1 − (1 − k_HB_* · *t)^n^]*	*	0.9679 *k_HB_ = 7.0 × 10^−6^* *n = 1111*	**0.9942** *k_HB_ = 2.4 × 10^−5^* *n = 145*	**0.9924** *k_HB_ = 0.012* *n = 1.227*
	**	**0.9924** *k_HB_ = 0.20* *n = 0.38*	0.9615 *k_HB_ = 2.0 × 10^−5^* *n = 175*	0.9449 *k_HB_ = 2.8 × 10^−5^* *n = 611*
**Baker-Lonsdale** *3/2* · *[1 − (1 − F/100)^2/3^] − F/100 = k_BL_* · *t*	*	0.9694 *K_BL_ = 3.3 × 10^−4^*	0.9588 *K_BL_ = 7.6 × 10^−5^*	0.9384 *K_BL_ = 1.5 × 10^−3^*
	**	0.9280 *K_BL_ = 1.4 × 10^−4^*	**0.9973** *K_BL_ = 2.9 × 10^−5^*	***0.9827*** *K_BL_ = 5.7 × 10^−4^*
**Peppas-Sahlin** *F = k_1_* · *t^m^ + k_2_* · *t^(2^*^∗*m)*^	*	**0.9935** *k_1_* = *0.290* *k_2_ = 0.545* *m = 0.553*	***0.9875*** *k_1_* = *0.621* *k_2_ = 0.060* *m = 0.638*	***0.9809*** *k_1_* = *3.349* *k_2_ = 0.302* *m = 0.573*
	**	**0.9929** *k_1_* = *0.800* *k_2_ = 0.094* *m = 0.148*	**0.9985** *k_1_* = *1.059* *k_2_ = 0.254* *m = 0.030*	**0.9999** *k_1_* = *2.665* *k_2_ = 0.072* *m = 0.030*
**Weibull** *F = 100* · *{1 − Exp[−((t − Ti)^β)/α^]}*	*	***0.9812*** *α = 42.564* *β = 0.714* *Ti = 1.517*	**0.9942** *α = 285.520* *β = 0.992* *Ti = 1.651*	**0.9925** *α = 13742.281* *β = 2.287* *Ti = 18.138*
	**	**0.9925** *α* = 644.845 *β* = 1.501 *Ti* = 3.204	**0.9975** *α* = 80.992 *β* = 0.532 *Ti = 2.1 × 10^−7^*	**0.9968** *α* = 5.794 *β* = 0.641 *Ti* = 1.801
**Gompertz** *F = 100* · *Exp{−α* · *Exp[−β* · *log(t)]}*	*	***0.9893*** *α* = 6.865 *β* = 1.053	***0.9895*** *α* = 8.882 *β* = 0.937	0.9623 *α* = 68.730 *β* = 3.013
	**	***0.9801*** *α = 6.973* *β = 1.014*	**0.9946** *α = 4.544* *β = 0.360*	**0.9924** *α = 3.433* *β = 0.702*

*—70 days; **—20 days.

**Table 5:** Results on ACHT encapsulation in various polyester microparticles. The conditions are given in Table 3; refer to the entries for lecithin-containing systems except PLLA.

Polymer	*EE* (%)	*DL* (μg/mg)	$\bar{D}$, μm (SEM)
PDLLA	61 ± 6	244 ± 25	2.5 ± 0.8
PLLA **^a^**	30 ± 4	120 ± 17	1.5 ± 0.5
PCL	25 ± 2	99 ± 8	3.5 ± 1.4
PPDL	5 ± 1	18 ± 5	1.1 ± 0.3

**^a^** Obtained using dichloromethane/acetone as the organic phase + Lutrol F98 + SDS as the surfactant system.

## Data Availability

The data are available within the article and its Supplementary Materials.

## Supplementary Materials

The following supporting information can be downloaded at: https://www.mdpi.com/article/10.3390/polym17192676/s1. Figure S1: The graphical representation of protein release profiles approximation with various mathematical models.

## Abbreviations

The following abbreviations are used in this manuscript:

PDLLA	Poly(D,L-lactide)
PLLA	Poly(L-lactide)
PCL	Poly(ε-caprolactone)
PPDL	Poly(ω-pentadecalactone)
BSA	Bovine serum albumin
ACHT	α-Chymotrypsin
SEM	Scanning electron microscopy
DLS	Dynamic light scattering
SEC	Size-exclusion chromatography
EE	Encapsulation efficacy
DL	Drug loading
DMAB	Didodecyldimethylammonium bromide
ROP	Ring-opening polymerization
