# Supplementary material for: Comparison of Different Aliphatic Polyester-Based Microparticles as Protein Delivery Systems
Source: Polymers (Basel). 2025 Oct 3;17(19):2676. doi: 10.3390/polym17192676 (PMC12526760; doi:10.3390/polym17192676)
Supplement: Supplementary file 1 [file polymers-17-02676-s001.zip › polymers-3894407-supplementary.pdf]

polymers

MDPI

Supplementary Materials

# Comparison of Different Aliphatic Polyester-Based Microparticles as Protein Delivery Systems

**Viktor Korzhikov-Vlakh [1,*], Ekaterina Sinitsyna [1,2], Mariia Stepanova [2], Evgenia Korzhikova-Vlakh [1,2] and Tatiana Tennikova [1]**

[1] Institute of Chemistry, Saint-Petersburg State University, 198504, St. Petersburg, Russia; kat_sinitsyna@mail.ru (E.S.); e.korzhikova-vlakh@spbu.ru (E.K.-V.); tennikova@mail.ru (T.T.)
[2] Branch of Petersburg Nuclear Physics Institute Named by B.P. Konstantinov of National Research Center "Kurchatov Institute"—Institute of Macromolecular Compounds, 199004, St. Petersburg, Russia; maristepanova@gmail.com
* Correspondence: v.korzhikov-vlakh@spbu.ru

**Zero-order** $F=k_{zo}*t$

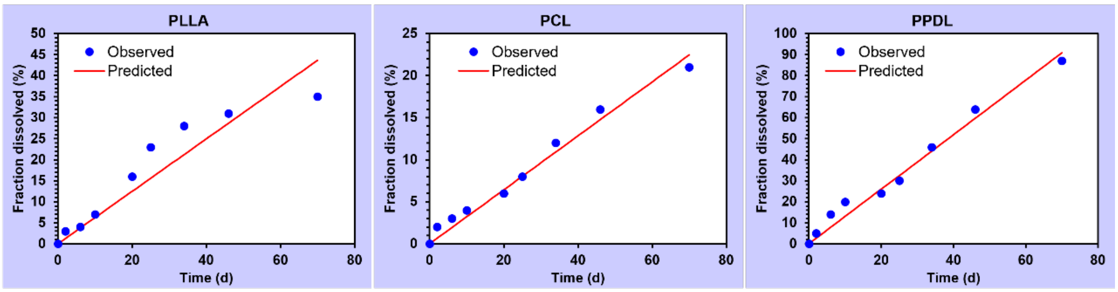


**First-order** $F=100*[1-Exp(-k_{fo}*t)]$

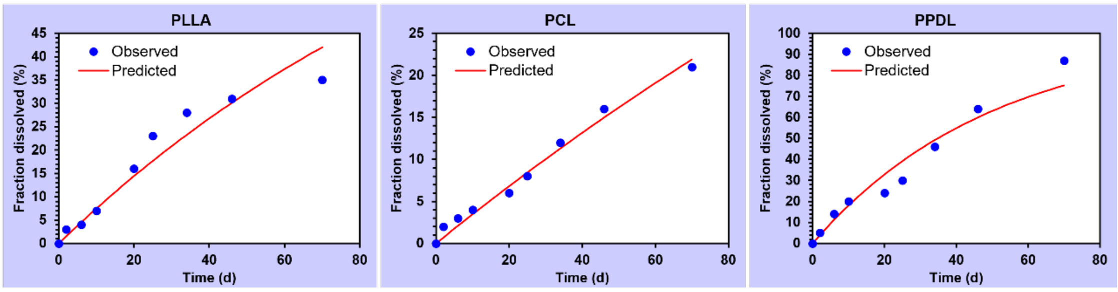


**Higuchi** $F=k_H*t^{0.5}$

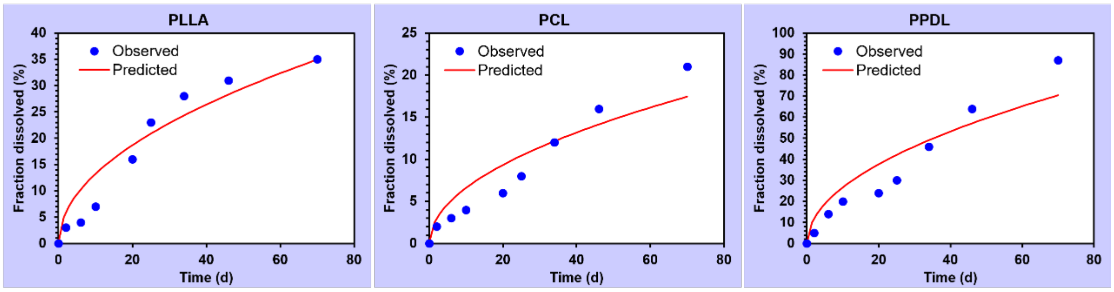


**Korsmeyer-Peppas** $F=k_{KP}*t^n$

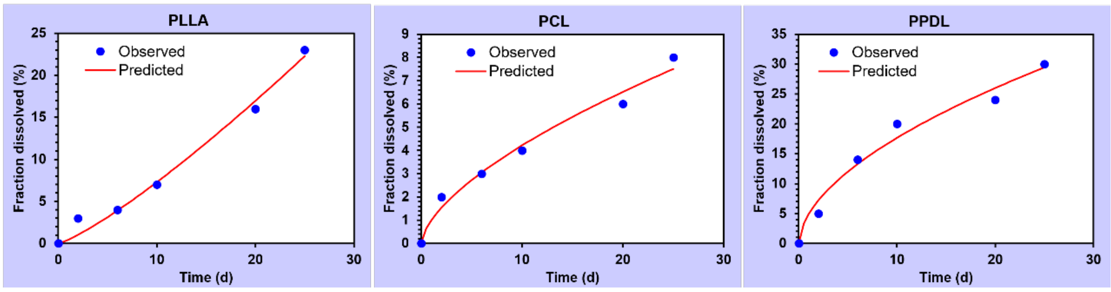

**Hixson-Crowell** $F=100*[1-(1-k_{HC}*t)^3]$

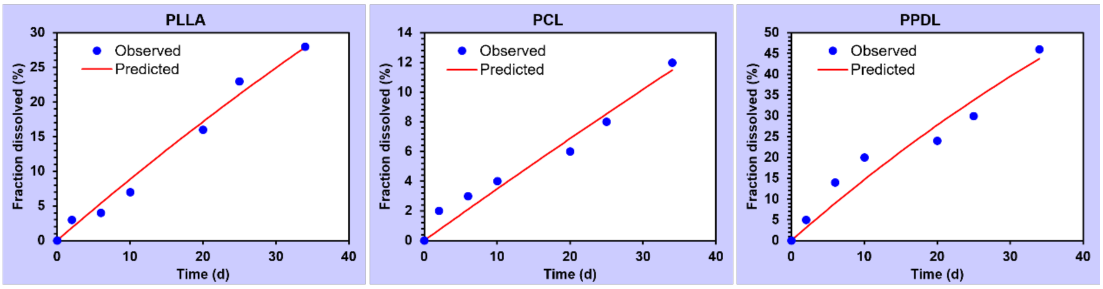


**Hopfenberg** $F=100*[1-(1-k_{HB}*t)^n]$

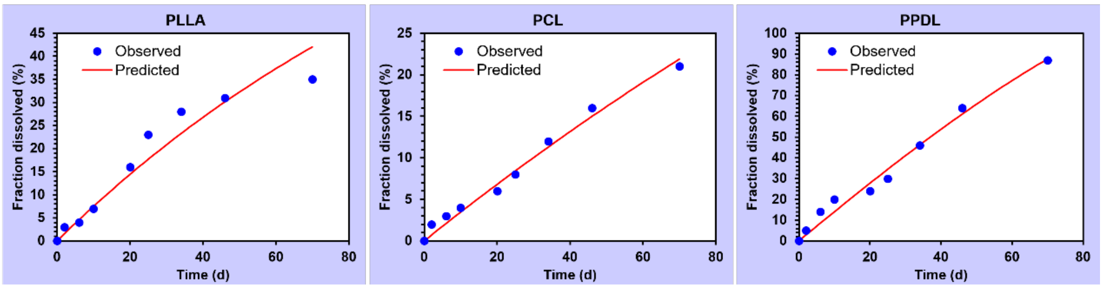


**Baker-Lonsdale** $3/2*[1-(1-F/100)^{(2/3)}]-F/100=k_{BL}*t$

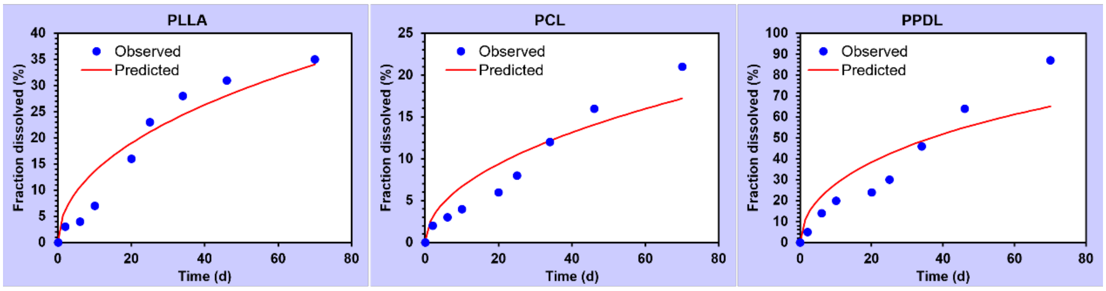


**Weibull** $F=100*\{1-Exp[-((t-Ti)\text{^}\beta)/\alpha]\}$

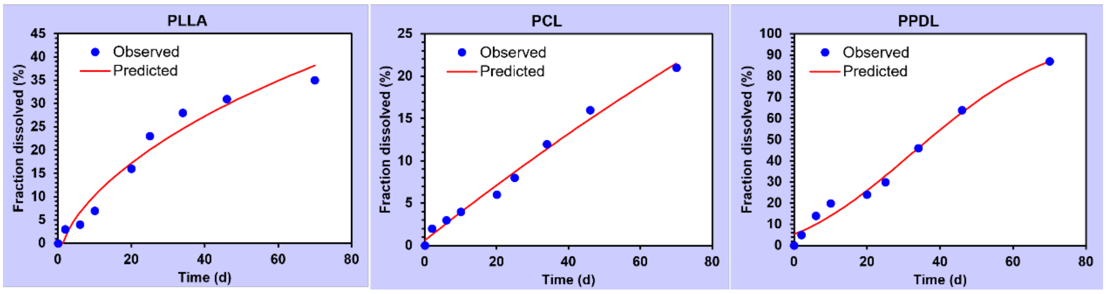


**Gompertz** $F=100*Exp\{-\alpha*Exp[-\beta*log(t)]\}$

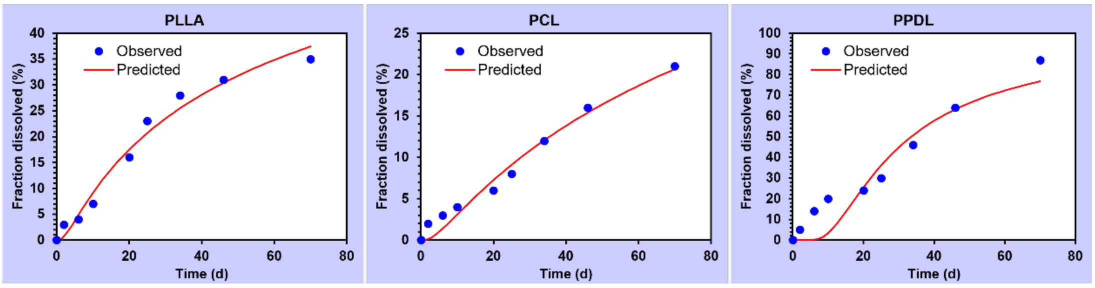


**Figure S1.** The graphical representation of protein release profiles approximation with various mathematical models.
